# The Use of Artificial Neural Networks in Prediction of Congenital CMV Outcome from Sequence Data

**DOI:** 10.4137/bbi.s764

**Published:** 2008-05-29

**Authors:** Ravit Arav-Boger, Yuval S. Boger, Charles B. Foster, Zvi Boger

**Affiliations:** 1 Department of Pediatrics, Division of Infectious Diseases, Johns Hopkins Hospital, Baltimore; 2 Optimal Neural Informatics, Pikesville; 3 Department of Pediatrics, Division of Infectious Diseases, The Cleveland Clinic, Cleveland, OH

## Abstract

A large number of CMV strains has been reported to circulate in the human population, and the biological significance of these strains is currently an active area of research. The analysis of complex genetic information may be limited using conventional phylogenetic techniques.

We constructed artificial neural networks to determine their feasibility in predicting the outcome of congenital CMV disease (defined as presence of CMV symptoms at birth) based on two data sets: 54 sequences of CMV gene UL144 obtained from 54 amniotic fluids of women who contracted acute CMV infection during their pregnancy, and 80 sequences of 4 genes (US28, UL144, UL146 and UL147) obtained from urine, saliva or blood of 20 congenitally infected infants that displayed different outcomes at birth. When data from all four genes was used in the 20-infants’ set, the artificial neural network model accurately identified outcome in 90% of cases. While US28 and UL147 had low yield in predicting outcome, UL144 and UL146 predicted outcome in 80% and 85% respectively when used separately. The model identified specific nucleotide positions that were highly relevant to prediction of outcome. The artificial neural network classified genotypes in agreement with classic phylogenetic analysis. We suggest that artificial neural networks can accurately and efficiently analyze sequences obtained from larger cohorts to determine specific outcomes.\

The ANN training and analysis code is commercially available from Optimal Neural Informatics (Pikesville, MD).

## Introduction

CMV is a ubiquitous virus that infects the majority of humans by adulthood.^1^ CMV infection is also the most common congenital infection in the U.S., affecting around 1% of infants.^2^ While most CMV-infected infants have no symptoms at birth, approximately 10% are severely symptomatic and have major central nervous system complications including hearing loss, seizures and mental retardation.^3^,^4^ Hearing loss develops in the majority of symptomatic infants but also in some previously-asymptomatic ones. These variable outcomes involve viral and host determinants. The role of specific CMV strains and/or infection with multiple CMV strains in the outcome of congenital CMV infection is unclear.^5^,^6^

Most of the 165 genes present in the 236 kbp human CMV genome are highly conserved among strains. However, at least 12 genetic loci are unusually variable (4–15 subsets in each) and sequencing at multiple genetic loci reveals that a large but finite number of strains is in circulation.^7^ The number of strains continues to increase because of the lack of intra- and inter-genic linkage.^8^

The most popular approach for analyzing sequence variation and genetic clustering is the phylogenetic analysis. Several studies have reported associations between specific CMV strains and disease outcome, defined as presence of CMV symptoms at birth.^6^,^9^ However, considering the hypervariability of several genes, phylogenetic analysis may be limited in determining associations between polymorphisms and outcomes, as well as in identifying genetic substitutions across multiple loci. The genetic information obtained from studies of strain variation in different populations can be extensive because of the number of subjects and the number of genetic loci that are sequenced. Therefore, more tools are in need to analyze complex and multiple genetic factors. The ability to predict the outcome of congenital CMV infection based on virus heterogeneity may have major clinical significance.

Artificial neural networks (ANNs) have been successfully applied in different fields to address complex problems. ANNs learn by an iterative process that adjusts the weights of the connections between the artificial neurons, such that the system outputs an appropriate result. Data processing by these systems does not require assumptions of how outputs relate to inputs. Similarly successful learning does not require independent inputs. As such, ANN analysis is sometimes able to confirm causal input-to-output relationships that were discovered using traditional means, as well as uncover additional insight and knowledge.

Because of the high sequence variability in several CMV genes (e.g. 70% divergence at amino acid level for UL146),^10^ and the many different genetic strains that were observed, we decided to construct an ANN model, to determine whether we could enhance prediction accuracy of congenital CMV disease outcome based on sequence data from four CMV-encoded genes: UL144, UL146 and UL147 and US28. In addition, we determined the ability of the ANN model to identify specific nucleotide positions that were particularly related to outcome.

## Materials and Methods

### Samples

We obtained samples from two cohorts: 54 amniotic fluids from Italian women and 23 cultured samples (urine, saliva or blood) from the US-born neonates diagnosed with congenital CMV. Thirteen viral isolates (8 urine samples, 4 saliva samples and one blood sample) were cultured from symptomatic neonates, ten other isolates (all saliva samples) were cultured from asymptomatic, CMV-infected neonates. Our previous work indicated that there is no geographically-related difference in the genotype distribution between the United States and Europe.^11^ Additionally, our experience shows that, in a specific CMV-infected infant, the same DNA sequences are detected from different body fluids.

### DNA extraction and genotyping

Total genomic cell and viral DNA was extracted from infected cells and original amniotic fluids using a capture-column kit (Gentra systems, Minneapolis, Minnesota). PCR amplification of US28, UL144, UL146 and UL147 was described elsewhere.^6^,^10^ Out of 23 samples obtained from US born neonates, DNA amplification for US28, UL144, UL146 and UL147 was successful in 20 samples (7 asymptomatic, 13 symptomatic infants) and provided 80 sequences total. PCR products obtained from both cohorts were sequenced directly with the BigDye Terminator Cycle Sequencing Kit (Perkin-Elmer Applied Biosystems, Foster City, California) and the sequencing products were analyzed on an ABI 310 automated sequencer. Sequence alignment and phylogenetic analyses were described elsewhere.^6^ Multiple alignment of coding DNA from aligned amino acid sequences was done using RevTrans.^12^

### Design of ANN models

An ANN model (also called ‘Multilayer Perceptron’) was constructed from three layers of mathematical “neurons”: input layer, a single hidden layer, and output layer. The output of each neuron is a function of the values of the inputs to it multiplied by calculated weights of each input. ANN models are trained by learning from known examples, and adjusting the weights between the neurons so that the errors between the ANN outputs and the known data are minimized. After training of the ANN, additional information is obtained by analyzing the individual weights that connect the input layer into the hidden layer, and the hidden layer into the output layer,^13^ and inputs with the greatest impact on outputs can be determined. Starting non-random weights were calculated using published algorithm.^14^ Five hidden neurons were used. Once the ANN was trained, we tested the sensitivity and accuracy of the model to identify specific outcomes based on sequences presented as validation samples that were not used in the training process. The outcome of the patients was already known to us but the ANN network performed “blinded” analysis after its training was completed. Furthermore, the training and validation samples were randomly selected from the entire data set, and selection was not based on outcome.

### Construction of inputs to the ANN

We combined the sequence data of UL144, UL146, UL147, and the N-terminus region of US28 into one vector for each of the samples. Each vector had 1631 nucleotide positions: 531 positions for UL144, 377 for UL146 (after alignment), 483 for UL147 and 240 for US28. These 20 vectors were then transformed into 20 binary vectors, by expanding each nucleotide position into four binary positions. “A”, “C”, “G”, and “T” were coded as [1 0 0 0], [0 1 0 0], [0 0 1 0] and [0 0 0 1] respectively. Positions with nucleotide deletions were coded as [0 0 0 0]. This binary coding resulted in binary vectors with 1,631 × 4 = 6,524 positions. Binary positions that were identical among all 20 samples were removed, resulting in a set of 1451-long binary vectors comprised of 301 inputs for UL144, 827 for UL146, 268 for UL147 and 55 for US28. The higher position count for UL146 is indicative of the hypervariability of this gene. For the 54-sample group that only had UL144 data, a similar process was repeated, resulting in a 301-long binary vector for each sample.

### ANN output

The ANN was constructed with a single output, designating outcome at birth (severe congenital CMV disease or asymptomatic CMV infection with no sequelae at birth). Samples from symptomatic infants were denoted as an output value of 0.9 whereas asymptomatic samples were denoted as an output value of 0.1. Both hidden and output neurons used the sigmoid function.

$$\sigma (y)=\frac{1}{1+e^{-y}}$$

Where *y* is the sum of individual input values *x_j_* times the individual weights connecting the inputs to the neuron:

$$y=\sum_{i=1}^{e}w_{ij}x_{i}$$

Training was done on a notebook computer with a 1.66 GHz Intel Core Duo processor (IBM Think-Pad 2623D4U) using Matlab numerical analysis software (Mathworks, Natick, MA) with ANN training and analysis code from Optimal Neural Informatics (Pikesville, MD). The training is stopped when the sum of all squared errors of each output neurons for the training examples (not the validation examples) does not decrease by at least 5% for 9 consecutive iterations (including iterations where the local minima escape algorithm was invoked).

### Training methods

We first analyzed the 54 samples with UL144 data. 36 samples were randomly selected for training, and the remaining 18 samples were selected for validation. The ANN was trained, optimizing the individual weights connecting input to hidden and hidden to output so that the ANN outputs were as close as possible to known outcomes. Thereafter, the ANN model was tested for its ability to predict the outcome of the other 18 samples which were not used as part of the training. An ANN output of 0.5 or higher, was defined “symptomatic”, and an ANN of less than or equal to 0.5 was defined as “asymptomatic”. The quality of the network was determined using two measures: 1) the number of samples which were correctly predicted and 2) the AUC (area under curve) of the ROC (receiver operating curve). In a perfectly-accurate model, AUC would be 1. This process was repeated 100 times with a different random assignment of samples into the validation and training groups in each time.

When considering the 20-sample data set, we realized that training the ANN with just 12 samples might be difficult. Thus, we employed the commonly-used “leave one out” training strategy, as follows: we performed 20 “leave one out” training sessions. In each session, a different sample was used as an independent test, while the remaining 19 out of the 20 samples were used as training vectors. Once trained, the ANN was tested by predicting the outcome of the independent test sample which was not used as part of the training.

To determine the most significant nucleotides in each gene that predict outcome, the “leave one out” process was repeated using data from only one gene at a time, as well as various combinations of two genes. To verify the validity of the “leave one out” method, we also performed it on the 54-sample Italian cohort, and compared the results with those obtained when taking 36 samples for training and 18 for validation.

In the 20-sample cohort we determined the specific nucleotide positions in each of the four tested genes that were most relevant to the network in determining outcome. To find these locations, we randomly divided the samples into two groups: 15 samples (5 asymptomatic, 10 symptomatic) for training and 5 samples (2 asymptomatic, 3 symptomatic) for testing. We then ranked the inputs based on their relevance to the ANN prediction accuracy, calculated using the hidden neurons relative variance (HDRV) knowledge extraction and dimensionality reduction technique.^13^ This technique is based on the observation that in a trained ANN model, a less relevant input contributes only a small proportion of the variance in the activities of hidden neurons. After each iteration, we removed the least significant inputs that contributed a total of 10% of the variance of the hidden neuron, according to their causal index (CI) as defined below, and left those inputs that contributed 90% of the variance. We then re-trained the network using the same 15 training samples but with the reduced input set and repeated this process of training and input removal 20 times.

We calculated the causal index (CI) of each input, a semi-quantitative estimate of the direction and magnitude of the influence of each ANN input on the ANN output.^15^ For any combination of input neuron *i* and output neuron *k*, the causal index is defined as

$${CI}_{ik}=\sum_{j=1}^{h}w_{kj}w_{ij}$$

Where *h* is the number of hidden neurons, *w_kj_* are the connection weights from hidden neuron *j* to output *k*, and *w_ij_* are the connection weights between input *i* and hidden neuron *j*. The CI was found to be very useful in relating the influence of change in each input to the relative magnitude and direction change of each output.^15^ The magnitude of the CI estimates the relative contribution of each input to the output value. The sign of each coefficient (positive or negative) estimates in which direction does the input affect the output value. Large positive CI means that a particular input strongly influences the outcome towards being symptomatic (0.9 output value), whereas large negative CI means that a particular input strongly influences the outcome of the network towards an asymptomatic outcome (0.1 output value). Although somewhat heuristic, the CI is more reliable than local sensitivity analysis as it is based on data from the entire ANN using all the available states.

Last, we analyzed the output of the hidden neurons in network trained with all 20 samples. It has been published^16^ that in a well-trained ANN, these outputs tend to be close to 0 or 1. For each input sample, we rounded the value of the each hidden neuron to 0 or 1 (using a 0.5 threshold) and used these “binary” patterns of the hidden layer for each set of inputs can be used for clustering of input vectors into similar groups.

## Results

### Italian cohort (54 samples with UL144 data)

We randomly selected 36 samples to train an ANN and 18 samples to validate it. We then recorded the total number of correct classifications in the validation group as well as the total number of correct classification in the training group. We then repeated this process 100 times, each time performing a different random selection to the training and validation groups. On average, 13.4 samples (74.4%) were correctly classified from the validation group, with a standard deviation of 1.75 samples (9.7%). Out of the entire 54-sample cohort, 48.9 samples (90.5%) were classified correctly on average, with a standard deviation of 2.3 samples (4.3%). Mean AUC for validation group was 0.88, with a standard deviation of was 0.07.

The “leave one out” analysis correctly predicted 83% of the samples (11 of 16 symptomatic, 34 of 38 asymptomatic) with an AUC of 0.88.

### 3.2 US cohort (20 samples analyzed with UL144, UL146, UL147 and US28)

Using all 4 genes, ANNs predicted outcome in 90% of the samples (6 out of 7 asymptomatic, 12 out of 13 symptomatic). Table 1 summarizes the results of ANN prediction of outcome. Only two samples (A6 and S11) were incorrectly identified. ROC analysis revealed an AUC of 0.857.

**Figure 1:**
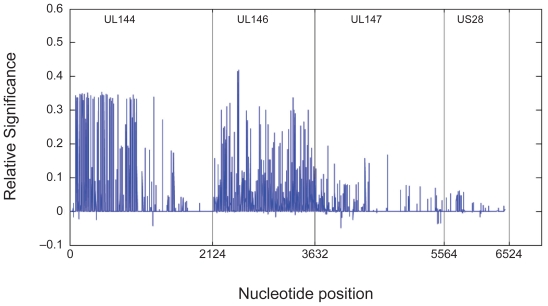
Relevance values for UL144, UL146, UL147 and US28. (Positions that were eliminated in the preprocessing are shown as 0).

We determined the relative relevance (HDRV) index for each of the nucleotide position (Fig. 1). Most of the relevant values are concentrated in the UL144 and UL146 regions. Summarizing the absolute value of the relevance for each gene shows that UL146 contained 48.2% of the total relevance, UL144 contains 43.4%, UL147 contains 7.1% and US28 contains 1.4%. Thus, UL147 and US28 appear insignificant in determining outcome.

To further test the hypothesis that UL144 and UL146 were most relevant to prediction of outcome, we performed ANN analysis when using only one gene or a combination of several genes at a time (Table 2). The “leave one out” ANN prediction for various gene combinations revealed that the highest prediction accuracy (85%) was achieved when using UL146 data alone or UL146 data in combination with UL147 or US28 data. Comparing UL144-based networks with UL146-based networks revealed that prediction accuracy with UL144 is slightly lower (80% vs. 85%), but AUC is slightly higher (0.824 vs. 0.791). Thus, prediction based on UL144 is very similar to prediction based on UL146, and the differences may be reduced with a larger data set. As expected, UL147 or US28 alone produced inaccurate networks as evidenced by both low prediction accuracy and low AUC (for US28).

**Figure 2:**
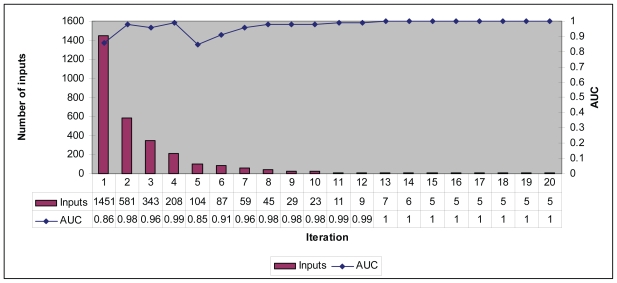
Number of remaining inputs and area under the curve (AUC) for each iteration of the input-reduction algorithm.

20 iterations of the input count reduction procedure were executed (Fig. 2) with a resultant AUC.

The positions chosen from the 9th iteration onwards are shown in Table 3. Prediction accuracy for the 9th and 11th iteration was 90%, including 100% of the samples that did not participate in the input reduction process. Prediction accuracy for the 10th and 12th iteration was 95%, including 100% of the samples that did not participate in the input reduction process. Inputs identified in the 13th iteration onwards were able to accurately predict 100% of the samples.

We performed clustering of input samples by training an ANN with UL144 inputs. The network identified the following three clusters: Cluster 1: samples A1, A4, A5, A6, A8 and A10 (all asymptomatic), cluster 2: samples S1, S2, S5, S6, S8, S10, S11, S12, S13 and S14 (all symptomatic), cluster 3: samples S3, S4 and S7 (all symptomatic). These clusters are comparable to the phylogenetic clustering previously published on these samples^6^. Repeating the process using only the UL146 inputs, we identified three clusters: cluster 1: samples A1, A4, A5, A8 and A10 (all asymptomatic), cluster 2: samples A6, S1, S2, S3, S4, S5, S6, S7, S8, S10, S12, S13 and S14 (all symptomatic), cluster 3: Samples A9 and S11.

## 4. Discussion

We report that ANN is a sensitive and effective method for the analysis of complex CMV polymorphisms, prediction of outcome of CMV infection and knowledge extraction. There is an ongoing debate whether strain variation plays a role in CMV disease outcome in general and congenital CMV in particular. Sequencing of several gene loci reveals high degree of sequence variation, but the biological significance of this finding is unclear. The comparison between and the definition of genotype at each locus is largely based on divergence levels and clustering, which are distinct for each gene, and give unambiguous results. We have reported that polymorphism in the CMV-encoded UL144, a truncated TNF receptor gene, was predictive of the outcome of congenital CMV infection among 23 US and 56 Italian newborns.^6^,^11^ Polymorphisms in UL146 and UL147, both α-chemokine genes, were not found to be associated with disease severity among 23 US newborns.^10^ High degree of sequence variation was noted in UL146, and therefore segregation of genotypes among asymptomatic or symptomatic newborns was technically impossible, and especially so given the sample size. Analysis of an association between gene polymorphisms and disease outcome is performed for one gene at a time. Thus, if the outcome depends on several genes, than identifying correlation between genetic clusters and outcomes becomes even more difficult.

The ability to predict symptomatic CMV disease from DNA sequence data is important, because it may allow early diagnosis and therapeutic considerations. Therefore a system that is able to analyze concurrently complex and high number of strains may advance our ability to predict outcome and detect more virulent strains. In fact, the artificial neural network approach has been applied to predicting CMV disease after renal transplantation, and revealed that the predictions were a considerable improvement on current prediction methods available at that time, although viral loads have not been used as parameters in this model.^17^

An ANN analyzes data when the relationships between cause and affect are complex and unclear. ANN modeling has been successfully used in many different fields, including medicine and biology.^18^ Specifically, ANN has been used in bio-modeling,^19^ and in molecular sequence analysis.^20^ Researchers have used ANN modeling for analysis of gene expression arrays of cancer cells^16^,^21^,^22^,^23^ identifying several genes that can correctly classify cancer types. In addition, ANN modeling has been used to enhance prediction of Lopinavir resistance from HIV genotype.^24^

The ANN method does not pre-suppose any knowledge about the relationship between inputs and outputs. In principal, we can use any combinations of genes as a possible predictor of disease outcome, and use insights derived from ANN analysis as triggers for more detailed studies using standard molecular laboratory techniques.

Even without prior knowledge, the ANN model generated results that are in agreement with our previously-published findings obtained using phylogenetic analysis and clustalW methods. The ANN modeling accurately predicted outcome of congenital CMV disease based on UL144 sequences. However, using the conventional phylogeny, we were unable to find a significant association between UL146 polymorphisms and outcome of congenital CMV, largely due to extreme hypervariability and a small sample size. When applying ANN modeling for prediction of congenital CMV outcome based on UL146 sequences, UL146 genotypes were predictive of outcome, while in UL147 and US28 no positions were found that correlated with disease outcome. In addition, a combination of four genetic loci was 90% sensitive in predicting outcome of congenital CMV.

Our analysis on the larger Italian cohort showed that the “leave-one-out” methods correlates with results obtained using the traditional ANN approach (2/3 of samples for training, 1/3 of samples for validation). This correlation further supports the use of the “leave-one-out” approach for the smaller US cohort. We attribute the somewhat better results obtained with the “leave-one-out” method to the larger sample size available for training: 53 samples in the leave-one-out method vs. 36 samples in the traditional approach. Both approaches generated very similar AUC values.

Using an ANN modeling also allowed us to determine the inputs (nucleotide positions) that have the most significant effect on the output (disease outcome). The model extracted the minimum number of nucleotide changes that resulted in the best prediction. While the ANN did not need more than 5 inputs to make a perfect prediction for the sample, we decided to provide additional inputs that were still present in late-stage iterations as they may lead to more focused questions related to biological mechanisms.

Clustering using ANN modeling was also analyzed. The fact that automatic ANN clustering based on UL144 matched our previous findings using more conventional techniques helped validate the automatic method. Since clustering for UL146 data was very difficult with conventional techniques (because of high degree of variability), we found it interesting that clustering could be performed with the ANN. Similarly, if samples need to be clustered based on more than one gene, an ANN is capable of producing relevant results whereas conventional distance-based methods face greater difficulties.

In summary, we have shown the potential use of analyzing large sequence information using ANNs modeling in addition to conventional phylogentic techniques. The ANN seems to have several advantages over regular phylogenetic analysis including concurrent analysis of multiple genetic loci, clustering into subtypes and identifying the most significant positions that affect the output. We recognize that the analysis was performed on a small sample size and a limited number of hypervariable genes, yet results are intriguing. At this time, we do not have sequence information on other hypervariable CMV genes such as gN and gB. Future studies should include a larger cohort of samples and sequence data of other hypervariable CMV genes. This will allow us to determine the role of different CMV strains in outcome of congenital CMV. We may also be able to create a prediction model that links different inputs (such as nucleotide sequences, multiple CMV strains, race, age, and family history) to outputs (such as symptomatic or asymptomatic disease, hearing loss etc). The successful development of such a model will allow identification of groups at higher risk for disease sequelae.

## Figures and Tables

**Table 1:** Prediction of congenital CMV outcome based on an ANN model using sequence data from 4 CMV-encoded genes. Bold numbers include incorrect classification.

Asymptomatic expected result = 0.1)		Symptomatic (expected result = 0.9)	
Sample	ANN prediction result	Sample	ANN prediction result
A1	0.21	S1	0.91
A4	0.16	S2	0.93
A5	0.35	S3	0.88
A6	**0.91**	S4	0.71
A8	0.09	S5	0.53
A9	0.49	S6	0.9
A10	0.2	S7	0.9
		S8	0.93
		S10	0.99
		S11	**0.14**
		S12	0.92
		S13	0.82
		S14	0.89

**Table 2:** Prediction performance (accuracy and AUC) of CMV outcomes based on various gene combinations.

Genes analyzed	Prediction accuracy	Correctly classified	AUC
		A: Asymptomatic	
		S: Symptomatic	
UL144, UL146, UL147, US28	90%	A:6/7, S: 12/13	0.857
UL146, UL147	85%	A:5/7, A:12/13	0.824
UL146, US28	85%	A:5/7, A:12/13	0.824
UL146	85%	A: 5/7, S: 12/13	0.791
UL144	80%	A: 4/7, S: 12/13	0.824
UL144, UL147	75%	A: 5/7, S: 10/13	0.824
UL147	75%	A: 3/7, S: 12/13	0.802
UL 144, UL146	70%	A: 4/7, S: 10/13	0.802
UL147, US28	70%	A:4/7, S:10/13	0.769
UL144, US28	60%	A: 3/7, S: 9/13	0.725
US28	55%	A:1/7, S: 10/13	0.495

**Table 3:** Specific important inputs identified by ANN. Causal index (9th iteration) is also reported to show magnitude of direction of influence for each input. Note: Causal Index for later iterations was identical in direction for each input and very similar in relative magnitude.

					Input reduction iteration						
Gene	Nucleotide	Value	Amino acid	Causal index	9	10	11	12	13	14	15–20
UL144	56	A	19	−6.82	✓	✓					
	66	A	22	−7.08	✓	✓					
	72	A	24	−6.81	✓	✓					
	108	C	36	−6.90	✓	✓	✓				
	115	A	39	−6.94	✓	✓					
	116	A		−6.82	✓	✓					
	118	C	40	−7.12	✓	✓					
	119	A		−7.01	✓	✓					
	126	T	42	−6.94	✓	✓					
	140	A	47	−7.01	✓	✓					
	180	T	60	−7.23	✓	✓	✓	✓	✓		
	226	G	76	−6.94	✓	✓	✓				
	234	T	78	−6.94	✓	✓					
	298	T	100	−10.27	✓	✓	✓	✓	✓	✓	✓
UL146	9	A	3	−9.25	✓	✓	✓	✓			
	46	A	16	8.22	✓	✓	✓	✓	✓	✓	✓
	46	G		−8.37	✓	✓	✓	✓	✓	✓	✓
	70	A	24	6.70	✓						
	96	T	32	−5.60	✓						
	140	G	47	9.00	✓						
	140	T		−10.6	✓						
	207	A	69	6.59	✓	✓	✓	✓	✓	✓	✓
	227	A	76	−8.13	✓	✓					
	262	C	88	−5.51	✓	✓					
	302	G	101	−5.35	✓						
	303	A		8.10	✓	✓	✓	✓			
	304	A	102	6.04	✓	✓	✓	✓	✓	✓	✓
	355	C	119	5.81	✓	✓	✓	✓	✓	✓	
UL147	51	T	17	4.88	✓						

## Abbreviations

ANN: artificial neural network
CMV: cytomegalovirus
PCR: polymerase chain reaction
